# Stereoselective Cytotoxicity of Enantiopure 3,3-Dichloro-γ-lactams: Correlating In Vitro Activity with In Vivo Tolerability

**DOI:** 10.3390/molecules30244778

**Published:** 2025-12-15

**Authors:** Faïza Diaba, Elisabet Teixidó, María del Carmen Morán

**Affiliations:** 1 Laboratori de Química Orgánica, Facultat de Farmàcia i Ciències de l’Alimentació, Institut de Biomedicina de la Universitat de Barcelona (IBUB), Universitat de Barcelona, Av. Joan XXIII 27-31, 08028 Barcelona, Spain; 2 GRET-Unitat de Toxicologia, Departamento de Farmacología, Toxicología y Química Terapéutica, Facultat de Farmàcia i Ciències de l’Alimentació, Institut de Recerca en Nutrició i Seguretat Alimentària (INSA), Universitat de Barcelona, Av. Joan XXIII 27-31, 08028 Barcelona, Spain; eteixido1511@ub.edu; 3 Departament de Bioquímica i Fisiologia-Secció de Fisiologia, Facultat de Farmàcia i Ciències de l’Alimentaciò, Universitat de Barcelona, Av. Joan XXIII 27-31, 08028 Barcelona, Spain; mcmoranb@ub.edu; 4 Institut de Nanociència i Nanotecnologia—IN2UB, Universitat de Barcelona, Av. Diagonal, 645, 08028 Barcelona, Spain

**Keywords:** enantiopure γ-lactams, in vitro cytotoxicity, cancer, in vivo Zebrafish, hemocompatibility, selective toxicity, 3T3, HaCaT, A431, HeLa, MCF-7

## Abstract

Enantiopure γ-lactams have emerged as promising scaffolds for anticancer drug development, yet the influence of stereochemistry on their biological activity remains insufficiently explored. In this study, we evaluated the in vitro cytotoxicity and in vivo toxicity of selected enantiopure 3,3-dichloro-γ-lactams and their corresponding epimers, with particular focus on their selectivity toward tumor versus non-tumor cells. The compounds were synthesized through green photoredox processes and tested for cytotoxicity using MTT and NRU assays in human cancer cell lines (A431, HeLa, MCF-7) and non-tumor controls (3T3, HaCaT). Toxicity and tolerability were further assessed in vivo using zebrafish (Danio rerio) embryos. Among the series, compound **3** displayed the most favorable selectivity index, combining potent anticancer effects with reduced activity toward non-tumor cells (HaCaT). Consistent with the in vitro results, compound **3** also demonstrated superior tolerability in zebrafish embryos compared with compound **4**. These findings highlight the critical role of stereochemistry in modulating the cytotoxic and safety profiles of γ-lactams. Overall, compound **3** ((*R*)-3,3-dichloro-4-methyl-1-((*S*)-1-phenylethyl)pyrrolidin-2-one) emerges as a promising candidate for further preclinical development.

## 1. Introduction

**Figure 1:**
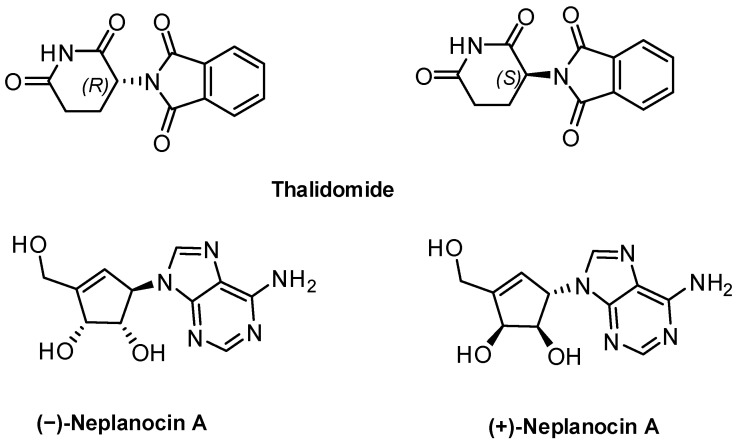
Examples of enantiomers exhibiting distinct biological activities.

Chirality is intrinsic to biology. Enzymes, receptors, nucleic acids, and membranes are themselves chiral and often distinguish between enantiomers of a molecule. As a result, two mirror-image forms or enantiomers of a bioactive compound can differ dramatically in binding affinity, uptake, metabolism, toxicity, and efficacy. This stereochemical discrimination means that the enantiopure form of a compound often exhibits superior therapeutic properties compared to the racemate or the other enantiomer [1,2,3]. A classic and tragic example highlighting the critical importance of chirality in drug development is thalidomide. Initially marketed in the late 1950s as a racemic drug to treat morning sickness and anxiety in pregnant women, thalidomide’s enantiomers were later found to have strikingly different biological activities. While the (*R*)-enantiomer was responsible for the intended sedative activity, the (*S*)-enantiomer caused severe teratogenic effects, leading to widespread birth defects [4]. More recently, Pawlowska et al. conducted a comparative study to evaluate the cytotoxicity of the two enantiomers of neplanocin A against a panel of cancerous and non-cancerous cell lines [5]. The results revealed that the naturally occurring (−)-enantiomer exhibited significantly higher cytotoxicity than the (+)-enantiomer across all tested cell lines (Figure 1).

**Figure 2:**
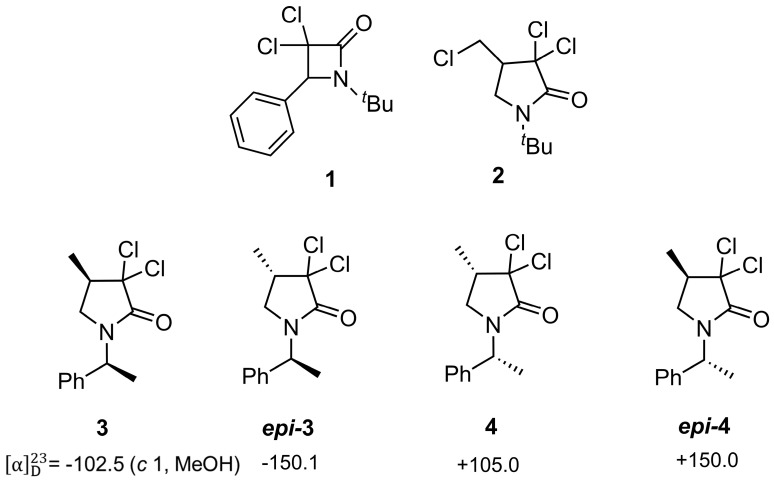
Previously Reported (**1**,**2**) and Newly Investigated Molecules (**3**,**4**).

In a recent study, we investigated ten 3,3-dichlorolactams (β-, γ-, and δ-lactams) for anticancer potential through hemocompatibility, cytotoxicity, and zebrafish embryo toxicity assays [6]. All compounds were non-hemolytic, but cytotoxicity varied depending on structure and dose. Among the investigated structures, β-lactam **1** (Figure 1) stood out as the most active and selective, showing strong cytotoxicity against A431 tumor cells (IC_50_ ≈ 35–71 µg/mL) while sparing normal 3T3 fibroblasts (IC_50_ > 210 µg/mL), with a selectivity index up to >7. Mechanistic studies confirmed apoptosis as its primary mode of action. In vivo, β-lactam **1** displayed lower acute toxicity than some anticancer drugs, though it caused developmental and neurotoxic effects in zebrafish embryos at concentrations below those effective in cancer cells, highlighting both its promise and safety limitations. In another work, we also found that compound **2** displayed the lowest IC_50_ values (approximately 100–250 µg/mL across different cell lines) and showed promising selectivity for A431 cancer cells compared to 3T3 non-tumor fibroblasts [7]. In both investigations, the tested molecules were in racemic form (Figure 2), suggesting that further studies with the individual enantiomers could provide deeper insight into their cytotoxic profiles and potential selectivity.

As a starting point for this investigation, we decided to evaluate the biological activity of the enantiopure lactam **3**, and its epimer ***epi*-3**, which share structural similarities with compound **2**. Both **3** and ***epi*-3** had been previously prepared in our research group within the framework of another synthesis project [8]. Moreover, to further investigate the role of stereochemistry in modulating biological function, the corresponding enantiomers, **4** and ***epi*-4**, were also synthesized and analyzed (Figure 2).

## 2. Results and Discussion

### 2.1. Synthesis of Lactams **4** and **epi-4**

**Scheme 1:**
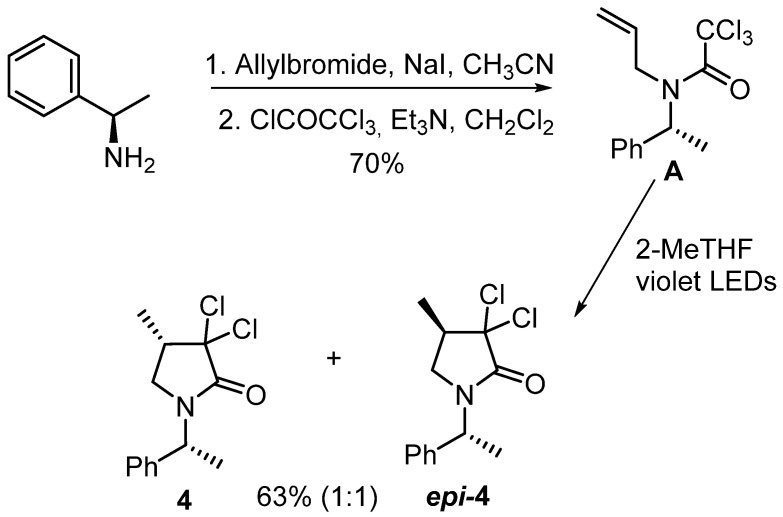
Preparation of **4** and ***epi*-4** from (*R*)-(+)-1-phenylethylamine.

As stated earlier, with **3** and ***epi*-3** in hand, we next prepared **4** and ***epi*-4** using the same three-step sequence applied in our prior work [8]. Thus, alkylation of (*R*)-(+)-1-Phenylethylamine with allyl bromide in the presence of sodium iodide provided the corresponding secondary amine which was treated with trichloroacetyl chloride in the presence of triethylamine to furnish trichloroacetamide **A** with a good yield. **A** was then submitted to ultraviolet irradiation in 2-Methyltetrahydrofuran to give **4** and ***epi*-4** in an acceptable yield (Scheme 1). The NMR spectra of **4** and ***epi*-4** were identical to those of their previously reported enantiomers [8].

### 2.2. Hemocompatibility Studies

According to EU regulations (ISO 10993-4) [9], the biocompatibility of new compounds that may come into contact with blood must be evaluated through In Vitro hemocompatibility testing. In this study, the hemolytic potential of compounds **3**, ***epi*-3**, **4** and ***epi*-4** was assessed by incubating them with an erythrocyte suspension at two concentrations (10 and 80 µg/mL). The results demonstrated that all tested compounds were completely non-hemolytic, with hemolysis values below 2% in all cases, indicating their potential suitability for further development as safe blood-contacting agents [10].

### 2.3. Cell Viability Assays

Following the criteria of our previous work, for cytotoxicity evaluation, two non-tumor cell lines were included as controls: 3T3, a mouse embryonic fibroblast line widely used as a model of normal cells, and HaCaT, an immortalized but non-tumor human keratinocyte line representative of healthy skin. The cytotoxic activity of the compounds was then assessed against several human cancer cell lines: A431, derived from epidermoid carcinoma of the skin and frequently employed in epidermal growth factor receptor (EGFR) related studies; HeLa, the well-established cervical cancer cell line; and MCF-7, a breast adenocarcinoma line that is estrogen receptor–positive and commonly used in breast cancer research.

**Figure 3:**
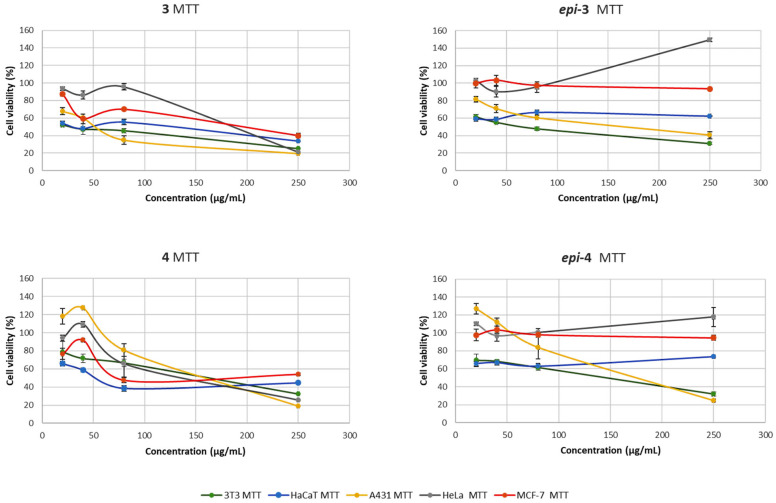
Concentration-dependent viability of 3T3, HaCaT, A431, Hela and MCF-7 cells treated with lactams **3**, ***epi*-3**, **4** and ***epi*-4** for 24 h, determined by MTT.

Two different endpoints, 3-(4,5-dimethyl-2-thiazolyl)-2,5-diphenyltetrazolium bromide (MTT) and neutral red uptake (NRU), were used to assess differences in cell-induced cytotoxicity. MTT offers details about the modification of the metabolic activity of the mitochondria inside the cells upon incubation with drugs [11]. In addition, NRU evaluates the interaction of these drugs with the plasmatic membrane [12]. Cell responses were determined from the MTT and NRU assays using the proposed cell lines. The cytotoxic activity of compounds **3**, ***epi*-3**, **4**, and ***epi*-4** was evaluated by MTT assay on a panel of cancer cell lines (A431, HeLa, and MCF-7) and two non-tumor controls (3T3 and HaCaT). Cytotoxicity assays were performed at concentrations of 20, 40, 80 and 250 µg/mL. When the cytotoxic response was evaluated by MTT (Figure 3), compound **3** exhibited a clear dose-dependent reduction in cell viability across all tumor cell lines, with the strongest effect observed in A431 and HeLa cells. Notably, compound **3** maintained significant activity at higher concentrations, while its effect on non-tumor 3T3 and HaCaT cells was less pronounced, suggesting a degree of selectivity toward malignant cells. In contrast, ***epi*-3**, its diastereomer, showed minimal cytotoxicity since the viability of most cell lines remained close to or above 80% even at the highest tested concentration, indicating that the epimerization at C-4 led to a drastic loss of activity. Compound **4**, enantiomer of **3**, also reduced viability in a dose-dependent manner, although its activity appeared slightly lower compared to **3**. Compound **4** showed higher toxicity toward non-tumor cells, particularly 3T3, which may limit its selectivity. Finally, ***epi*-4** behaved similarly to ***epi*-3**, with poor activity against cancer cell lines and little evidence of a dose-dependent response. Overall, these results highlight that stereochemistry plays a critical role in the cytotoxic properties of these γ-lactams, the enantiopure forms (**3** and **4**) are significantly more potent than their corresponding epimers (***epi*-3** and ***epi*-4**).

**Figure 4:**
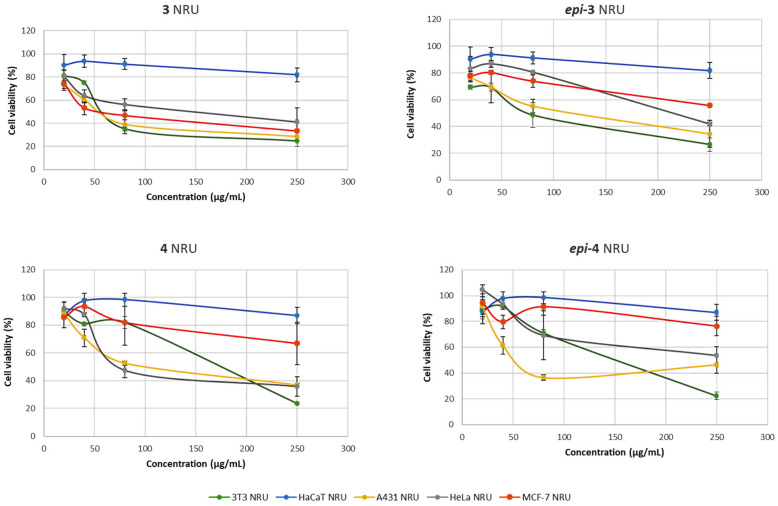
Concentration-dependent viability of 3T3, HaCaT, A431, Hela and MCF-7 cells treated with lactams **3**, ***epi*-3**, **4** and ***epi*-4** for 24 h, determined by NRU.

The NRU assay confirmed the trends observed in the MTT experiments (Figure 4). Compound **3** demonstrated a strong, dose-dependent cytotoxic effect on all the cancer cell lines (A431, HeLa, and MCF-7), with cell viability dropping to around 20–30% at the highest tested concentration. Importantly, the compound exhibited only moderate effects on non-tumor HaCaT cells, reinforcing its targeted activity against malignant cells. By contrast, ***epi*-3** showed a markedly weaker response, although some reduction in viability was observed in A431 and HeLa cells, overall cytotoxicity remained limited, and cell survival in several lines stayed around 60% even at 250 µg/mL. Compound **4** also exhibited a clear cytotoxic profile, reducing viability across both cancerous and non-tumor cells in a concentration-dependent manner; however, its lower selectivity, particularly the stronger effect on 3T3 fibroblasts, may limit its therapeutic potential compared to compound **3**. Finally, ***epi*-4** produced only moderate cytotoxicity, with partial activity against tumor cells but reduced potency compared to its enantiopure counterpart. Generally, the NRU results corroborate the MTT data, highlighting that the enantiopure forms (**3** and **4**) are consistently more active than their epimers, and that compound **3** in particular shows the most favorable balance of potency and selectivity.

The results from both the MTT and NRU assays consistently demonstrated that stereochemistry plays a crucial role in the cytotoxic activity of the investigated γ-lactams. The enantiopure compound **3** and its enantiomer **4** exhibited pronounced, dose-dependent cytotoxicity against the tumor cell lines A431, HeLa, and MCF-7, while their epimers (***epi*-3** and ***epi*-4**) showed markedly reduced or negligible effects. Compound **3** emerged as the most promising candidate, combining strong activity against cancer cells with comparatively lower effects on the non-tumor controls (3T3 and HaCaT), thus indicating a degree of selectivity. In contrast, compound **4**, although cytotoxic, affected both cancerous and non-tumor cells suggesting that its stereochemical configuration confers reduced selectivity. The convergence of findings from two independent viability assays reinforces the robustness of these observations (Figure 3 and Figure 4). Overall, these findings imply that the structure of lactam **3** confers optimal interactions with molecular targets involved in cell viability, and that the stereochemical integrity of this scaffold is essential for maintaining cytotoxic function.

The corresponding half-maximal inhibitory concentration (IC_50_) was determined from the fitting of concentration-dependent viability curves. The Selectivity index (SI) as a measurement of the action against the tumor cell line in comparison with a non-tumor cell line was also included. Table 1 summarizes representative results under the assayed conditions. When the corresponding IC_50_ could not be determined, this value was considered to be higher than the highest tested concentration (250 µg/mL).

As it is reported in Table 1, compound **3** exhibited the lowest IC_50_ values across several cell lines, particularly in 3T3 (30.03 μg/mL, MTT) and A431 (56.01 μg/mL, MTT), suggesting a stronger cytotoxic response relative to its epimer ***epi*-3**, whose IC_50_ values were consistently higher (>68 μg/mL). This difference indicates that stereochemical inversion at C-4 substantially decreases activity, pointing to a stereoselective interaction with cellular targets. Interestingly, compound **4**, while also cytotoxic, demonstrated broader and less selective activity, affecting both tumor and non-tumor cells. Its IC_50_ toward 3T3 fibroblasts (94.6 μg/mL, MTT) and HaCaT keratinocytes (57.27 μg/mL, MTT) suggests a loss of selectivity compared to compound **3**, consistent with its stereochemical modifications. ***Epi*-4** displayed generally weaker activity, with IC_50_ values above 100 μg/mL in most cases, indicating that this stereochemical configuration further reduces cytotoxic potential. When comparing assay endpoints, the MTT assay generally produced lower IC_50_ values than the NRU method, reflecting greater sensitivity of mitochondrial activity measurements relative to lysosomal integrity under these conditions.

The selectivity indices (SI), calculated as the ratio of the IC_50_ in the non-tumor 3T3 cell line to the IC_50_ values in the cancer cell lines (A431, HeLa, MCF-7), provide a measure of each compound’s preferential cytotoxicity toward tumor cells. Altogether, as is reported in Table 2, most SI values are close to or below 1, indicating limited selectivity and suggesting that the cytotoxic effects are comparable between tumor and non-tumor cells. Among the tested molecules, compound ***epi*-4** displayed the highest SI values, particularly in the NRU assay, with SI (3T3/A431) = 2.48. Compound **4** also showed slightly elevated SI values in the NRU assay, reaching 2.23 for HeLa and 1.60 for A431, though remaining near or below 1 in MTT measurements. In contrast, compounds **3** and ***epi*-3** exhibited SI values consistently below 1 across all assays, reflecting lower selectivity and suggesting that these compounds affect cancer and non-tumor cells with similar potency. Moreover, the statistical analysis demonstrated significant differences for compound **3** compared with the rest of the analyzed compounds when considering the SI between 3T3 and MCF-7 cell lines. A similar pattern was observed for compound **4** when considering SI between 3T3 and MCF-7 cells, where its activity differed significantly from that of all other compounds. Finally, for ***epi*-4**, significant differences were detected relative to the remaining stereoisomers in the SI between 3T3 and A431 cell lines.

Meanwhile, the selectivity indexes (SI) against HaCaT calculated as IC_50_ (HaCaT)/IC_50_ (cancer cell) values were generally higher than those obtained with 3T3 cells, indicating improved selectivity toward tumor cells, particularly for compound **3**. In the NRU assay (Table 3), the data indicate that compounds **3** and ***epi*-4** exhibit the highest apparent selectivity, particularly, where several SI values exceed 4. Compound **3** stands out, showing SI (HaCaT/A431) > 4.15 and SI (HaCaT/MCF-7) > 4.16, suggesting a markedly stronger cytotoxic effect on cancer cells than on HaCaT cells. Compound ***epi*-4** also displayed high SI values (>4.05 for A431) but was less consistent across other cell lines due to missing data. Consequently, the NRU assay again produced higher SI values than MTT, consistent with its greater sensitivity to membrane damage and sublethal effects. Taken together, these results highlight compound **3** as the most promising and selective molecule, showing consistent preference for tumor cells while maintaining low toxicity toward HaCaT controls. Furthermore, the statistical analysis revealed significant differences for compound **3** compared with the other compounds when considering the SI between HaCaT and A431 cell lines. A similar trend was observed when considering HeLa cells, where compound **3** showed significant differences when compared with ***epi*-3** and compound **4**. Finally, in the case of MCF-7 cells, significant differences were detected between the two methods used.

### 2.4. The In Vivo Zebrafish Experiments

**Figure 5:**
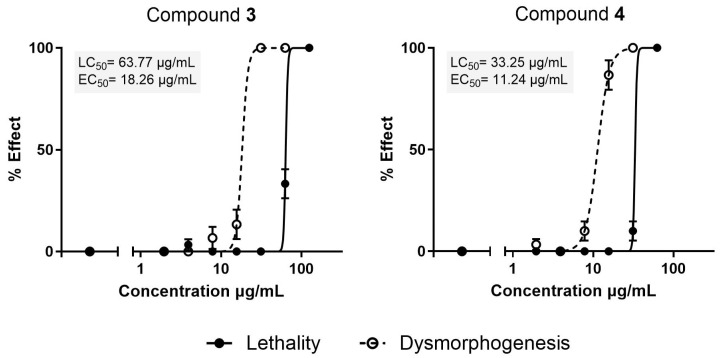
Concentration-response curves for lethality and dysmorphogenesis upon exposure to zebrafish embryos to compound **3** and **4** in the range 1.95–125 µg/mL. The results are shown as mean ± SEM of at least three independent experiments, using ten embryos for each concentration in each experiment. The lethal concentration 50 (LC_50_; concentration causing death in 50% of individuals) and the effective concentration 50 (EC_50_; concentration inducing dysmorphogenesis in 50% of larvae) were determined by fitting the mean values of the tested groups to a non-linear concentration–response curve with a variable slope using a least-squares method, setting the minimum and maximum responses at 0% and 100%.

The acute toxicity of compound **3** and **4** was evaluated using the Fish Embryo Acute Toxicity (FET) assay. Embryos were exposed from 2 to 72 hpf to increasing concentrations of compound **3** and **4** (from 1.95 to 125 µg/mL). Figure 5 shows the obtained lethal concentration 50 (LC_50_) and effective concentration 50 (EC_50_) values for compounds **3** and **4** after 72 h of exposure. In agreement with the In Vitro results, compound **4** exhibited greater toxicity in zebrafish embryos (LC_50_ = 33.25 µg/mL) compared to compound **3** (LC_50_ = 63.77 µg/mL), consistent with its lower IC_50_ value in HaCaT cells (57.27 µg/mL vs. 122.48 µg/mL obtained with the MTT method).

**Figure 6:**
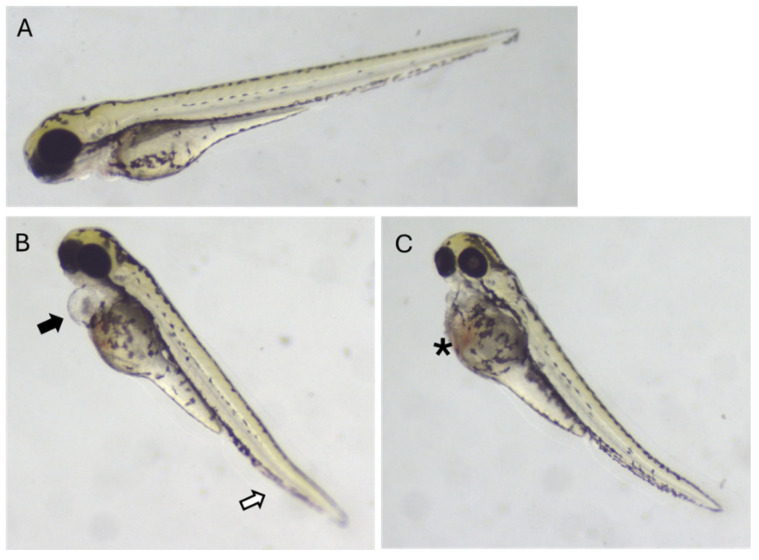
Representative morphological effects observed in zebrafish embryos at 74 hpf exposed to solvent control (1% DMSO) (**A**), 30.25 µg/mL of compound **3** (**B**) and 15.6 µg/mL of compound **4** (**C**). The black arrow indicates pericardial edema, the white arrow indicates flexion abnormality and the asterisk indicates hemorrhage.

Dysmorphogenic effects were produced at similar EC_50_ concentrations for compound **3** at 18.26 µg/mL and for compound **4** at 11.24 µg/mL. Morphological abnormalities mainly consisted of flexion abnormalities, pericardial edema and hemorrhages (Figure 6). The calculated Teratogenic Index (TI) values for compounds **3** and **4** were 3.5 and 2.95, respectively, both classified as potential teratogens (TI > 2). Both compounds showed low acute toxicity if compared to β-lactam **1** tested previously in zebrafish (LC_50_ = 14.9 µg/mL) [6].

**Figure 7:**
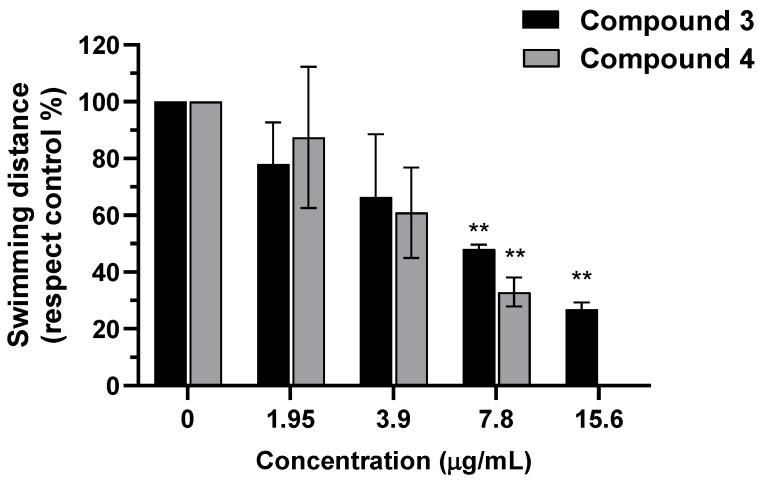
Evaluation of the touch-evoked response (TER) after exposure to compounds **3** and **4**. Results are expressed as the mean ± SEM of the swimming distance percentage relative to the control. The experiment was performed in triplicate, using six embryos per concentration group, with each embryo subjected to three tactile stimuli. For compound **4**, the 15.6 µg/mL concentration was excluded from the analysis, as more than 20% of the embryos exhibited malformations. Asterisks indicate a significant difference compared with the corresponding controls (** *p* < 0.01).

To assess neuromuscular function, we measured the response to a tactile stimulus (TER assay) after a 2 h washout period. A statistically significant neurodevelopmental adverse effect was observed in the TER from 7.8 µg/mL for both compounds. Compound **4** was evaluated only up to this concentration, as at 15.6 µg/mL more than 20% of embryos exhibited morphological abnormalities. No difference in neurotoxic effects was observed between compounds **3** and **4** (Figure 7).

Despite the observed differences in general toxicity, their neurobehavioral and morphological outcomes were essentially equivalent. Although no previous studies have evaluated the neurotoxic effects of anticancer enantiomers in zebrafish, enantioselective neurotoxicity has been reported for other classes of compounds, such as pesticides, where one enantiomer often exhibits greater developmental or neuronal toxicity than the other [13,14]. In contrast, our results show that both enantiomers of the tested γ-lactams produced similar neurotoxic outcomes, indicating that stereochemistry may not significantly influence their interaction with neuronal targets during early development.

## 3. Materials and Methods

### 3.1. Experimental Procedure for the Synthesis of Lactams **4** and **epi-4**

Preparation of trichloroacetamide **A** was carried out following the same two-step procedure from (*R*)-(+)-1-phenylethylamine that we reported previously [15]. A solution of **A** (200 mg, 0.65 mmol) in 2-MeTHF (13 mL) was stirred at rt under violet LEDs irradiation for 14 h. The reaction mixture was concentrated and purified by chromatography using a mixture of Hexane/EtOAc (100:0 to 90:10) to yield **4** and then ***epi*-4** (112 mg, 63%, 1:1 ratio). **4**: Physical state: colorless oil; ${\left[\alpha \right]}_{D}^{23}$ = +105.0 (*c* 1, MeOH); ^1^ H NMR (400, MHz CDCl_3_) δ 7.40–7.26 (m, 5H, ArH), 5.47 (q, *J* = 7.1 Hz, 1H), 2.98 (dd, *J* = 9.8, 7.1 Hz, 1H, H-5), 2.92 (dd, *J* = 9.8, 8.5 Hz, 1H, H-5), 2.59 (dquint, *J* = 8.4, 6.7 Hz, 1H, H-4), 1.54 (d, *J* = 7.1 Hz, 3H, CH_3_), 1.27 (d, *J* = 6.6 Hz, 3H, CH_3_); ^13^ C NMR (101 MHz, CDCl_3_) δ 166.6 (C-2), 138.7 (*ipso*-C), 128.8, 128.0, 127.1 (Ar-CH), 87.6 (C-3), 50.5 (CH), 45.4 (C-4 and C-5), 15.5 (CH_3_), 11.6 (CH_3_); IR (NaCl) 3059, 2978, 2937, 1713 cm^−1^; HRMS (ESI-TOF) calcd. for C_13_H_16_Cl_2_NO [M+H]^+^ 272.0603, found 272.0604. ***Epi*-4**: Physical state: white solid, m.p.: 89–91 °C; ${\left[\alpha \right]}_{D}^{23}$ = +150.0 (*c* 1, MeOH); ^1^ H NMR (400 MHz CDCl_3_) δ 7.38–7.25 (m, 5H, ArH), 5.49 (q, *J* = 7.1 Hz, 1H), 3.25 (dd, *J* = 9.9, 6.8 Hz, 1H, H-5), 2.73 (dquintet, *J* = 8.4, 6.7 Hz, H-4), 2.53 (dd, *J* = 9.9, 8.4 Hz, 1H, H-5), 1.58 (d, *J* = 7.1 Hz, 3H, CH_3_), 1.22 (d, *J* = 6.6 Hz, 3H, CH_3_); ^13^ C NMR (101 MHz, CDCl_3_) δ 166.6 (C-2), 138.5 (*ipso*-C), 128.7, 128.0, 127.0 (Ar-CH), 87.5 (C-3), 50.2 (CH), 45.4 (C-5), 45.3 (C-4), 15.8 (CH_3_), 11.8 (CH_3_); IR (NaCl) 3054, 2981, 2939, 1705 cm^−1^; HRMS (ESI-TOF) calcd. C_13_H_16_Cl_2_NO [M+H]^+^ 272.0603, found 272.0602.

### 3.2. Materials and Methods for In Vitro Assays

#### 3.2.1. Materials

Dulbecco’s modified Eagle’s medium (DMEM), fetal bovine serum (FBS), L-glutamine solution (200 mM), trypsin–EDTA solution (170,000 U/L trypsin and 0.2 g/L EDTA), penicillin–streptomycin solution (10,000 U/mL penicillin and 10 mg/mL streptomycin), and phosphate-buffered saline (PBS) were acquired from Lonza (Verviers, Belgium). MTT (3-(4,5-dimethyl-2-thiazolyl)-2,5-diphenyltetrazolium bromide) and neutral red dye (NR) were obtained from Sigma–Aldrich (St. Louis, MO, USA). T-75 flasks and 24- and 96-well plates were provided from TPP (Trasadingen, Switzerland). All other reagents were of analytical grade.

#### 3.2.2. Methods

##### In Vitro Assay Using Human Erythrocytes

Acquisition and Extraction of the Erythrocytes

Human blood samples were obtained from the Banc de Sang i Teixits de Barcelona (Barcelona, Spain) under the Catalan Department of Health. Blood was collected in tubes containing the anticoagulant EDTA-K3. Samples were centrifuged at 3000 rpm and 4 °C for 10 min (Megafuge 2.0 R, Heraeus Instruments, Hanau, Germany) to promote sedimentation, after which plasma was carefully eliminated using a Pasteur pipette. The remaining pellet was washed with PBS (pH 7.4) three times to remove residual leukocytes and platelets while concentrating erythrocytes. After the final wash, the erythrocyte pellet was resuspended in PBS (pH 7.4) at a 1:1 ratio to achieve a final erythrocyte concentration of 8 × 10^9^ cells/mL.

##### Hemolysis Assay

The hemolysis assay is used to evaluate the ability of the tested compounds to disrupt erythrocyte membranes. Stock solutions of each compound were prepared in PBS (pH 7.4) at a concentration of 1 mg/mL. Volumes ranging from 10 to 80 μL were transferred into polystyrene tubes, followed by the addition of 25 µL of the erythrocyte suspension, bringing the total volume to 1 mL. The samples were incubated at room temperature under gentle rotation and subsequently centrifuged at 10,000 rpm for 5 min. The absorbance of the resulting supernatants at 540 nm (Shimadzu UV-160A, Shimadzu, Duisburg, Germany) was measured and compared with that of the positive control (erythrocytes hemolyzed with distilled water) and the negative control (erythrocytes suspension in PBS, pH 7.4).

The degree of hemolysis was determined using the following equation:

Hemolysis (%) = 100 × (Abs − Abs0)/(Abs100 − Abs0)

where Abs, Abs0 and Abs100 are the absorbance of the test samples, of the suspension treated with isotonic phosphate–buffered saline (PBS) and of the suspension of complete hemolysis treated with distilled water, respectively.

##### In Vitro Assay Using Cell Cultures

The murine Swiss albino fibroblast (3T3), the immortal human keratinocyte (HaCaT), the squamous cell carcinoma (A431), the human epithelial carcinoma (HeLa) and the human breast adenocarcinoma (MCF-7) cell lines were sourced from Celltec UB (Barcelona, Spain). All cells were maintained and grown in DMEM medium (4.5 g/L glucose) supplemented with 10% (*v*/*v*) FBS, 2 mM L-glutamine, 100 U/mL penicillin and 100 μg/mL streptomycin at 37 °C, 5% CO_2_. Cells were routinely cultured in 75 cm^2^ culture flasks and were trypsinized using trypsin-EDTA when the cells reached approximately 80%. Cell concentration in the resulting suspension was assessed using the trypan blue assay, which enables direct discrimination between viable (unstained) and non-viable (blue) cells.

##### Cell Viability Assays

T3 and HaCaT cells (1 × 10^5^ cells/mL), along with A431, HeLa, and MCF-7 cells (5 × 10^4^ cells/mL), were cultured at defined seeding densities in the 60 central wells of a 96-well plate. Cells were first incubated for 24 h under standard incubator conditions (5% CO_2_, 37 °C). After this period, the culture medium was removed, and the cells were incubated for an additional 24 h with their corresponding compound solutions (1 mg/mL) previously diluted in a minimum amount of DMF (dimethylformamide) and then in DMEM medium supplemented with 5% FBS (100 µL) at the required concentration range (20 to 250 µg/mL). Cell viability following exposure to the lactam derivatives was evaluated using two complementary assays: NRU and MTT.

##### NRU Assay

The neutral red uptake (NRU) assay is based on the accumulation of dye in the lysosomes of viable cells. After the cells were incubated for 24 h with the corresponding systems, the medium was removed, and the solutions were incubated with the NR dye (Sigma-Aldrich, St. Louis, MO, USA) solution (50 µg/mL) dissolved in the medium, without FBS and without phenol red (Lonza, Verviers, Belgium), for 3 h. Cells were then washed with sterile PBS, followed by the addition of 100 µL of a solution containing 50% absolute ethanol and 1% acetic acid in distilled water to extract the dye. To promote the total dissolution of the dye, plates were placed in a microtiter-plate shaker for 5 min at room temperature. The absorbance of the resulting solutions was measured at 550 nm (Bio-Rad 550 microplate reader, Bio-Rad California, Hercules, CA, USA). Finally, the effect of each treatment was calculated as the percentage of dye uptake by viable cells relative to the control cells (cells without any treatment). Finally, the effect of each treatment was calculated as the percentage of dye retained by viable cells compared with untreated control cells.

##### MTT Assay

Only living cells can reduce the yellow tetrazolium salt 3-(4,5-dimethyl-2-thiazolyl)-2,5-diphenyltetrazolium bromide (MTT) to insoluble purple formazan crystals. After a 24 h incubation of the cells with their corresponding NPs, the medium was removed and 100 µL of MTT (Sigma-Aldrich, St. Louis, MO, USA) in PBS (5 mg/mL) diluted 1:10 in culture medium without phenol red and without FBS (Lonza, Verviers, Belgium) was added to the cells. After 3 h of incubation, the medium was removed. Thereafter, 100 µL of DMSO (Sigma-Aldrich, St. Louis, MO, USA) was added to each well to dissolve the purple formazan crystals. Agitation and absorbance measurements of the extracted solution were carried out under the same conditions described for the NRU assay. The effect of each treatment was then expressed as the percentage of tetrazolium salt reduction by viable cells relative to untreated controls.

##### Selectivity Towards Cancer Cells

The half-maximal inhibitory concentration (IC_50_) values for the different formulations, across cell lines and experimental endpoints, were obtained by fitting the concentration-dependent viability curves. For several cell lines, the IC_50_ of the tested compounds exceeded the highest concentration evaluated (250 µg/mL), indicating that 50% inhibition of cell viability was not reached under the experimental conditions. These findings reflect the very low cytotoxicity of the compounds toward these cell lines. When an IC_50_ could not be calculated, the value was reported as greater than the highest concentration tested (250 µg/mL).

The corresponding selectivity indexes toward tumoral cells were calculated using the following ratio:

SI = IC_50_ (non-tumor cell line)/IC_50_ (tumor cell line)

where 3T3 and HaCaT cells were used as representative non-tumor cell models.

### 3.3. Materials and Methods for In Vivo Assays

#### 3.3.1. Zebrafish Egg Production and Exposure

The study was conducted in accordance with the local legislation and institutional requirements according to the Generalitat de Catalunya Decree 53/2013, which regulates the use of animals for experimental and other scientific purposes.

Adult zebrafish, both male and female, were procured from a commercial supplier (Pisciber, Barcelona, Spain) and housed in a closed flow-through system with standardized dilution water, as specified in ISO 7346-1 [16] (2 mM CaCl_2_·2H_2_O; 0.5 mM MgSO_4_·7H_2_O; 0.75 mM NaHCO_3_; 0.07 mM KCl). The fish were maintained at a temperature of 26 ± 1 °C on a 14 h light and 10 h dark cycle and were daily fed in the morning with Artemia salina, and with commercial flake food in the afternoon (SDS400, Special Diet Services, Dietex, France). The day before eggs were needed, males and females were placed in a breeding tank with plants at a 2:1 male-to-female ratio. The following morning, eggs were collected 30 min after the lights were turned on. The eggs were then cleaned successively with reconstituted water according to ISO standard 7346-1. Fertilized, non-coagulated, and synchronously divided eggs were selected using a stereomicroscope (Motic SMZ168, Motic China group, Ltd., Xiamen, China) and transferred to 6-well plates (10 embryos/well). Embryos were exposed to freshly prepared tested concentrations of the compound in 0.3× Danieau’s solution. Chemical stock solution was prepared in 100% dimethylsulfoxide (DMSO) and subsequently diluted in Danieau’s buffer with a final DMSO concentration of 1% (*v*/*v*). Test was carried out using five concentrations with a negative control, solvent control with 1% of DMSO. Embryos were incubated at 27 ± 1 °C with a light–dark cycle of 14:10 h until 72 h post-fertilization (hpf) under semi-static conditions, as exposure solutions were renewed by freshly prepared solutions every 24 h.

#### 3.3.2. Evaluation of Lethality and Dysmorphogenesis

At 24, 48 and 72 hpf embryos were observed under a stereomicroscope to assess lethality. The lethality criteria, based on OECD Test Guideline No. 236 [17], included coagulation of embryos, absence of somite formation, and non-detachment of the tail starting from 24 hpf, with the addition of lack of heartbeat from 48 hpf onward [18]. At the end of the test, dysmorphogenesis was evaluated in live embryos only. The average lethality and dysmorphogenesis were calculated for 10 embryos per concentration, followed by averaging the results of at least three independent experiments. A sigmoidal (variable slope) curve fit was applied to these means to generate concentration-response curves and to calculate the LC_50_ (the concentration causing lethality in 50% of the larvae) and EC_50_ (the concentration causing dysmorphogenesis in 50% of the larvae). The Teratogenic Index (TI) was deter-mined as the ratio between the LC_50_ and EC_50_ (TI = LC_50_/EC_50_).

#### 3.3.3. Touch-Evoked Response (TER) Test

At 72 hpf, test solutions were replaced with 0.3× Danieau’s solution without the chemical and incubated at 27 °C ± 1 °C for 2 h. Subsequently, TER was conducted according to the protocol described by Guzman et al. [19]. In brief, videos were recorded using a Casio Exilim EX-ZR200 video camera (Tokyo, Japan) and the distance swam by zebrafish embryos was measured after three mechanical stimuli, applied to the tail with a forceps tip (Fine Science Tools, Dumont #5, Foster City, CA, USA) at 10 s intervals. The distance was averaged and converted from pixels to millimeters. This assay was performed only on larvae from concentration groups where both lethality and dysmorphogenesis were less than 20%. Six larvae per concentration group were evaluated in at least three independent experiments.

#### 3.3.4. Statistical Analysis

Statistical analysis was performed using GraphPad Prism v.10.0.2. TER test was analyzed using one-way ANOVA and the post-hoc multiple-comparison Dunnett test. Significance threshold was established at *p* < 0.05.

## 4. Conclusions

This work highlights the crucial influence of stereochemistry on the biological activity of 3,3-dichloro-γ-lactams. The enantiopure compounds **3** and its mirror image **4** exhibited pronounced cytotoxic effects, whereas their corresponding epimers were largely inactive, emphasizing the importance of stereochemical configuration in modulating bioactivity. Among the four lactams evaluated, compound **3** emerged as the most promising candidate, demonstrating potent and selective cytotoxicity toward cancer cells while sparing non-tumor controls, namely, HaCaT. In vivo assays in zebrafish further validated its superior tolerability relative to compound **4**, supporting its favorable safety profile. In summary, these findings position compound **3** as a strong lead molecule for future optimization and preclinical development aimed at advancing novel anticancer agents.

## Figures and Tables

**Table 1:** Cell viability assessment: half-maximal inhibitory concentration (IC_50_) values of the corresponding lactam derivatives **3**, ***epi*****-3**, **4** and ***epi*****-4** as a function of the cell line and endpoint method.

Cells	3 IC_50_ (μg/mL)		*epi*-3 IC_50_ (μg/mL)		4 IC_50_ (μg/mL)		*epi*-4 IC_50_ (μg/mL)	
	MTT	NRU	MTT	NRU	MTT	NRU	MTT	NRU
3T3	30.03	65.05	68.12	77.22	94.60	172.59	145.35	153.19
HaCat	122.48	>250	>250	>250	57.27	>250	>250	>250
A431	56.01	60.16	169.71	122.96	164.4	107.70	177.12	61.68
Hela	183.66	160.04	>250	213.96	94.30	77.46	>250	>250
MCF-7	192.79	60.00	>250	>250	77.80	>250	>250	>250

**Table 2:** Selectivity Indexes (SI) of Enantiopure γ-Lactams **3**, ***epi*-3**, **4** and ***epi*-4** Toward Cancer Cell Lines Using 3T3 as Non-Tumor Control.

Lactam	SI (3T3/A431)		SI (3T3/HeLa)		SI (3T3/MCF-7)	
	MTT	NRU	MTT	NRU	MTT	NRU
3	0.54	1.08 ^a^	0.16 **^*epi*-4^**	0.41	0.16	1.08 ^a,***epi*-3,4,*epi*-4**^
*epi*-3	0.40	0.63 **^3,4^**	<0.27 **^4^**	0.36	<0.27	<0.31 **^4,*epi*-4^**
4	0.58	1.60 ^a,**3**^	1.01 **^3,*epi*-4^**	2.23 ^a,**3,*epi*-4**^	1.22 ^a,**3,*epi*-3,*epi*-4**^	<0.69
*epi*-4	0.82 **^*epi*-3^**	2.48 **^3,4,*epi*-3^**	<0.58	<0.61	<0.58 **^3^**	<0.61

^a^ *p* < 0.05 indicates significant differences between methods for the same compound. **^compound^** *p* < 0.05 indicates significant differences between compounds for the same method.

**Table 3:** Selectivity Indexes (SI) of Enantiopure γ-Lactams **3**, ***epi*-3**, **4** and ***epi*-4** Toward Cancer Cell Lines Using HaCaT as Non-Tumor Control.

Lactam	SI (HaCaT/A431)		SI (HaCaT/HeLa)		SI (HaCaT/MCF-7)	
	MTT	NRU	MTT	NRU	MTT	NRU
3	2.19 **^*epi*-3,4,*epi*-4^**	>4.15 ^a,***epi*-3,4**^	0.67	>1.56 ^a,***epi*-3,4**^	0.64	>4.17 ^a^
*epi*-3	>1.47 **^4^**	>2.03 ^a,**4,*epi*-4**^	n.d.	>1.17	n.d.	n.d. ^b^
4	0.35	>2.32 ^a,***epi*-4**^	0.61	>3.23 ^a,***epi*-3**^	0.74	n.d.
*epi*-4	>1.41 **^4^**	>4.05 **^a^**	n.d.	n.d.	n.d.	n.d.

^a^ *p* < 0.05 indicates significant differences between methods for the same compound. **^compound^** *p* < 0.05 indicates significant differences between compounds for the same method. ^b^ n.d. Not determined.

## Data Availability

The data supporting this investigation is in the article and Supplementary Materials.

## Supplementary Materials

The following supporting information can be downloaded at: https://www.mdpi.com/article/10.3390/molecules30244778/s1, Video S1: Example video recording of touch-evoked response of control embryo. Video S2: Example video recording of touch-evoked response of embryo exposed to compound **3** (15.6 μg/mL). Video S3: Example video recording of touch-evoked response of embryo exposed to compound **4** (7.8 μg/mL).
